# Art Training in Dementia: A Randomized Controlled Trial

**DOI:** 10.3389/fpsyg.2020.585508

**Published:** 2020-12-15

**Authors:** Katherine G. Johnson, Annalise A. D’Souza, Melody Wiseheart

**Affiliations:** Department of Psychology, York University, Toronto, ON, Canada

**Keywords:** art, dementia, cognition, working memory, delayed recall

## Abstract

**Objectives:**

The present study explores the effect of visual art training on people with dementia, utilizing a randomized control trial design, in order to investigate the effects of an 8-week visual art training program on cognition. In particular, the study examines overall cognition, delayed recall, and working memory, which show deficits in people with dementia.

**Method:**

Fifty-three individuals with dementia were randomly assigned into either an art training (*n* = 27) or usual-activity waitlist control group (*n* = 26). Overall cognition and delayed recall were assessed with the Montreal Cognitive Assessment (MoCA), and working memory was assessed with the Backward Digit Span task.

**Results:**

There were no group differences in overall cognition, or working memory, while a difference in delayed recall was undetermined, based on post-test—pre-test difference scores. Groups were comparable at baseline on all measures.

**Conclusion:**

The measures of cognition, delayed recall, and working memory used in this study were not affected by an 8-week visual art training program.

**Clinical Trial Registration:**

www.ClinicalTrials.gov, identifier NCT03175822.

## Introduction

Dementia is a clinical syndrome associated with over 60 conditions (Kahn-Denis, 1997), all of which are characterized by a progressive decline in memory and cognitive functioning that is severe enough to cause a loss of daily functioning (Stewart, 2004). The question of how to cope with the effects of this debilitating disease is persistent. While the main treatment for dementia is currently pharmaceuticals, pharmaceutical treatments can be accompanied by non-trivial side effects (Hersch and Falzgraf, 2007). Thus, due to these complications, as well as the support for non-pharmacological treatments in dementia care (Caulfield, 2011), it is increasingly suggested that pharmacological treatments for dementia be employed as a secondary or co-existing approach to non-pharmacological treatments (Douglas et al., 2004; Caulfield, 2011; Camartin, 2015; Sauer et al., 2016). This study examines one non-pharmacological intervention, art training, as a potential method for reducing the rate of cognitive decline associated with dementia.

One key motivator for exploring art training as a dementia intervention is from the reported artistic potential of dementia patients (Kleiner-Fisman and Lang, 2004; Miller and Hou, 2004; Fornazzari, 2005; Shinagawa and Miller, 2014). It appears that the potential to create art does not diminish when an individual develops dementia. Instead, individuals who develop varying forms and severities of dementia display a remarkable ability to produce and participate in arts activities (Kleiner-Fisman and Lang, 2004; Mendez, 2004; Fornazzari, 2005; Van Buren et al., 2013; Schneider et al., 2019). These reports fit within the known progression of three of the most prevalent forms of dementia: typically, memory and executive function impairments occur first, while visuomotor and severe visuospatial deficits occur later (Perry and Hodges, 1999; Camic et al., 2014; Chancellor et al., 2014; Ehresman, 2014)^1^. This suggests that by targeting a skill that is unlikely to have decline—arts training—maintenance of a skill that is likely to show decline throughout the progression of dementia—cognition—may occur, although this claim has yet to be confirmed. Further, arts training would need to be a cognitive exercise for such an effect to be expected.

Since cognitive decline is a core feature of all forms of dementia, assessing the potential cognitive benefits of any dementia intervention is essential. From the mere appreciation of art (receiving and processing information), to the creation of art (processing, planning, manipulating, revising, recalling, and producing an artwork), to the communication that occurs between participants and group members and facilitators (sharing and updating information), art training is intuitively a cognitive exercise that requires multiple cognitive domains including recall (Baddeley and Logie, 1999; Bhattacharya and Petsche, 2002; Pérez-Fabello and Campos, 2007; Takahashi and Hatakeyama, 2011; Young, 2014; Windle et al., 2018a; Schneider et al., 2019). Further, while not yet supported by quantitative data, it has been suggested that working memory is a particularly relevant component of visual art participation: researchers suggest that the mental maintenance and manipulation of visual imagery is of exceptional relevance to artistic ability and production (Baddeley and Logie, 1999; Pérez-Fabello and Campos, 2007; Takahashi and Hatakeyama, 2011; Young, 2014). However, when considering people with dementia, research on the subject of cognition and art is largely contradictory: a large body of qualitative research reports improved cognition and memory for those who participated in art making or viewing programs (Kahn-Denis, 1997; Rentz, 2002; Kinney and Rentz, 2005; Parsa et al., 2010; Peisah et al., 2011; Camic et al., 2014; Cowl and Gaugler, 2014; Young et al., 2015; Sauer et al., 2016; Windle et al., 2018a), while only two randomized controlled trials (RCTs) exist and both have shown no such cognitive improvements in people with dementia (Rusted et al., 2006; Hattori et al., 2011).

The contradictory nature of the art and dementia literature is troublesome: qualitative research supports cognitive benefits while quantitative RCTs do not^2^. Lack of memory benefits using RCTs is exemplified by two previous studies (Rusted et al., 2006; Hattori et al., 2011). The first study is an RCT (*n* = 39) comparing a 12-week coloring group to a 12-week calculation drill active control group (Hattori et al., 2011). This RCT failed to find benefits of art therapy for logical memory (a subscale of the Wechsler Memory Scale revised; WMS-R) in people with dementia. The second RCT (*n* = 21), which compared a 40-week art therapy intervention to a 40-week usual activity (day center) active control, also failed to find short-term memory differences (via two subtests of the Rivermead Behavioral Memory Test) (Rusted et al., 2006). Alternatively, the present study utilizes two separate memory tasks to assess both working (the Backwards Digit Span) and delayed (the delayed recall subsection of the MoCA) memory—both of which are impacted by varying forms of dementia (Tuokko et al., 1991; Stewart, 2004; Fornazzari, 2005).

While there are few art and dementia RCTs, and none have supported the cognitive benefit of art for persons with dementia, dozens of quasi-experimental or observational studies have supported the beneficial connection between art and dementia. However, these studies have notable limitations, including lack of control groups, poorly reported art programming, inadequate methodological detail, and minimal experimental evidence (Locher, 2007; Chancellor et al., 2014; Windle et al., 2014, 2018a,b; Young et al., 2015; Matthews, 2016; Sauer et al., 2016). Importantly, RCTs are the gold standard for assessing causality, and are needed to establish whether art training causes the benefits seen in observational studies. To address these methodological issues, the present study used a randomized controlled design with a control group, detailed methods and procedures, rigorous experimental control, assignment concealment, and volunteer blinding. Additionally, following suggested guidelines and approaches, the present study utilized validated and reliable quantitative measures (Windle et al., 2018a).

Our objective is to explore the currently unclear relationship between cognition and art for those with dementia. We aim to resolve the confusion caused by the related literature’s qualitative and quantitative disconnect by addressing the limitations of previous studies and providing a rigorously designed community-based art training and dementia RCT. By utilizing art training—an area that is unlikely to be heavily affected by varying forms of dementia and that is arguably a cognitive exercise—we expect the experimental participants to demonstrate less decline in overall cognition, delayed recall, and working memory at post-testing.

## Materials and Methods

### Participants

**FIGURE 1:**
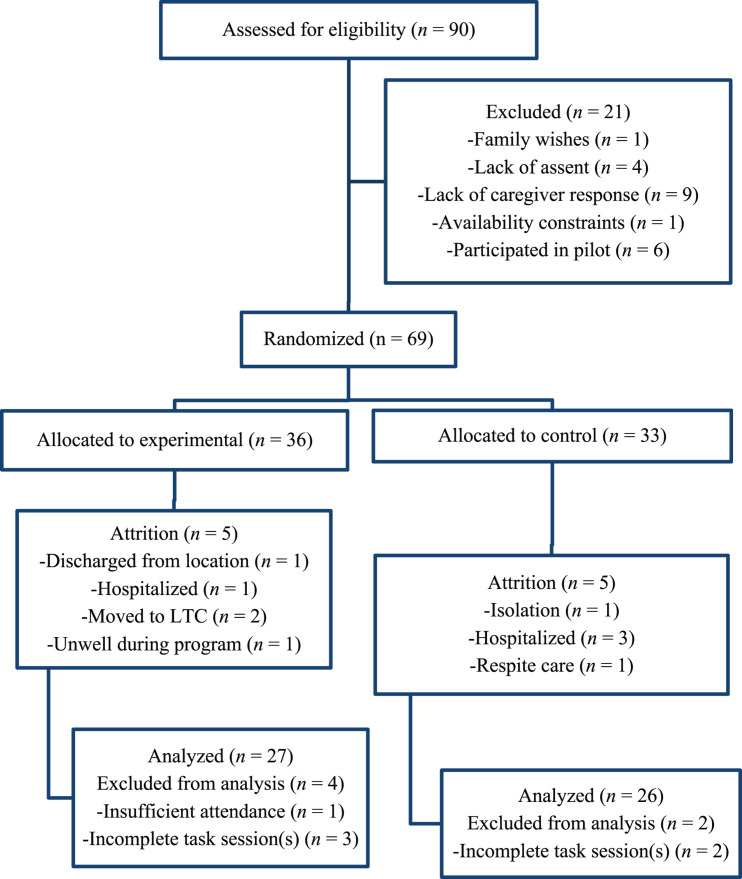
CONSORT diagram. LTC, long-term care.

A randomized controlled trial assessed two groups of dementia patients: an art training experimental group (*n* = 27) and a usual-activity waitlist control group (*n* = 26). After attrition, 53 individuals were included in the analyses (Figure 1)^3^. Participants were randomized into either group with a random number generator using simple randomization.

Recruitment for the project began May 2017. People with dementia were identified by recruited dementia care locations and were later verified via the Mini-Mental State Examination (MMSE; Folstein et al., 1975)^4^. Before a potential participant could participate, informative flyers and questionnaires were provided to each recruited location for distribution to participant families and caregivers. Substitute written informed consent was then provided by a potential participant’s respective care location via written consent for the project along with a list of approved individuals who could participate. Once potential participants were selected, they were each provided with an explanation of the study via a script and then asked, verbally, if they assented to participating (Figure 1).

Each participants’ tasks, intervention, and control activities were completed at their respective care facility. Caregivers did not participate in the task sessions or arts programs unless assistance was requested or required (e.g., in the event a participant was having language difficulties, tester volunteers could request assistance from a translator, fitting suggested guidelines) (King et al., 2015)^5^. This research project was approved by the York University Human Participants Research Sub-Committee, e2017-161, and registered at ClinicalTrials.gov (identifier: NCT03175822).

### Intervention

The present study’s art training program focused on actively learning art terminology and skills while providing participants with the materials and support to draw and collage. The terms that were focused on were the conceptual building blocks of visual art: the elements (space, color, texture, line, shape, form, and value) and principles (emphasis, variety, harmony, movement, rhythm, proportion, balance, and gradation) of design (see https://osf.io/dpc82/) (Foster, 2006).

Each art program occurred 1 h per day, 2 days per week, for 8 weeks, for a total of 16 sessions. This is in between the 12 and 40 sessions used in the two previous art-dementia RCTs (Rusted et al., 2006; Hattori et al., 2011). Further, while these RCTs failed to provide quantitative evidence (Rusted et al., 2006; Hattori et al., 2011), qualitative dementia research utilizing an 8-week timeframe have provided support for art’s beneficial effect on cognition (Camic et al., 2014, 2015; Young, 2014; Young et al., 2015). Each week focused on one specific activity, as suggested by the literature (Sauer et al., 2016), focusing on one element and one principle of design (Table 1). In addition to reflecting the related literature, the duration of the program (8 weeks, twice-per-week) was selected so that all eight principles and seven elements of design could be covered and focused on at least twice, while the 1-h duration was selected based on dementia location feedback and a review of the related literature (Rusted et al., 2006; Hattori et al., 2011). Each class offered an introduction (when the terms and activities of the day were explained), art-making (when the participants were invited to engage in the week’s activity), and interactive discussion (where willing participants could have their artwork shown to the group and relayed back to the week’s terms and activities) as suggested by the related literature (Flatt et al., 2015). Art programs were structured to capture the many factors involved in artistic activities: from those related to the physical artwork (e.g., balance, color, proportion) to the discussion of the physical artwork’s connection to artistic trajectories and personal and social contexts during group interaction and discussions (Ullán et al., 2013).

**TABLE 1:** Visual art intervention curriculum.

**Week**	**Term explanation**	**Activity**
One	Space: Positive and negative parts of an artwork that are distinct. Space can also provide depth in an artwork. Emphasis: Combining elements in a way that highlights the contrast between the elements.	White on Black Tracing: Instructors will bring in flat wooden objects, white and light-colored pencil crayons, and a stack of black paper. The paper will be placed in front of each participant’s seat, and the objects will be placed in the middle of the table. First, participants will be asked to choose an object that they like, then the instructor will instruct the participants to trace their respective objects onto the black paper and make a scene of their choosing with their traced shapes.
Two	Color: Consists of hue (name), value (lightness or darkness of the hue), and intensity (brightness and purity of the hue) Variety: Diversity and contrast of different elements.	Flat Object Coloring: Instructors will bring in flat wooden objects, markers and pencil crayons. The objects, markers and pencil crayons will be placed in the middle of the table. First, participants will be asked to choose an object that they like, then the instructor will instruct the participants to color in their objects. At the same time, participants will be encouraged to pass along an additional object, with each participant coloring in one area of the object at a time before passing it on to the next participant, and the next participant, and so on.
Three	Texture: The feeling of surfaces, or what a surface looks like it feels like. Harmony: The combination of similar elements within an artwork to highlight their similarities.	Fabric Collage: Instructors will bring in fabric, scissors, glue sticks, white paper, markers and pencil crayons. The white paper will be placed in front of each participant’s seat, and the fabric, glue sticks, markers and pencil crayons will be placed in the middle of the table. First, participants will be asked to choose fabrics they like, then the instructor will instruct the participants to make a picture with their selected fabric on their paper. Using markers and pencil crayons is also encouraged.
Four	Line: A mark moving in space. Lines can be literal or abstract. Movement: Creates action within an artwork and guides the art-viewer’s eyes through an artwork.	Tape Maze: Instructors will bring in line mazes, white paper, markers and pencil crayons. The mazes will be placed in front of each participant’s seat, and the white paper, markers and pencil crayons will be placed in the middle of the table. First, participants will be asked to move their finger or a pencil/marker through the maze, then instructors will instruct the participants to create their own line drawings on the separate pieces of white paper. Continuing to draw on the maze is also acceptable.
Five	Shape: A two-dimensional image. Rhythm: Repetition of elements in an artwork that creates visual movement, tempo, or beat.	Making shapes with Shapes: Instructors will bring in flat paper shapes, glue sticks, white paper, and markers and pencil crayons. The white paper will be placed in front of each participant, and the paper shapes, glue sticks, markers and pencil crayons will be placed in the middle of the table. Participants will be asked to use the shapes to create images (such as houses, butterflies, etc.) on the white paper.
Six	Form: A three-dimensional image which includes height, width and depth. Proportion: The relationship of elements to the entire artwork and to each other.	Object and People Proportion: Instructors will bring in white paper, props, markers and pencil crayons. The white paper will be placed in front of each participant’s seat, and the props, markers and pencil crayons will be placed in the middle of the table. To discuss proportion, the instructor and their class volunteer will stand together in front of the group. Once there, the instructor will ask the volunteer to stand behind them, then in front of them. The instructor will then use the props to show the same principle. Then, instructors will ask participants to draw proportion-related scenes on white paper. Props may be used for tracing.
Seven	Form: A three-dimensional image which includes height, width and depth. Balance: Combining elements to create equilibrium in an artwork.	Flat Image Team Organization: Instructors will bring in white paper, one large poster board, form stickers, markers and pencil crayons. A black line down the middle will split the poster board into two halves. All items, except the white paper, will be placed in the middle of the table. The instructors will then ask each half of the table (one half on one side of the board, the other on the other side) to take turns using the form stickers to “balance” the board (e.g., if the right-side places a large pink sticker on their side, the left-side places a large pink sticker on their side). Participants will match the other group’s sticker choice (based on color, shape or size) before choosing their own. The sticker balancing activity will go on until the board is filled. Participants will then be given paper and asked to draw similar “balanced” images on the white paper.
Eight	Value: The lightness and darkness of tones and colors. Gradation: The gradual change of elements.	Example Copying: Instructors will bring in markers, pencil crayons, white paper, and white paper with shapes on them; half with examples of value gradation (a square and circle that transitions from black to white) and half with empty shapes (an empty square and circle). The white paper with shapes will be placed in front of each participant, and the white paper, markers and pencil crayons will be placed in the middle of the table. Instructors will then ask participants to fill in the blank shapes like the gradation examples provided (gradation examples can also be colored).

Artworks were offered back to the participant and/or participant’s respective location at the end of the program, following protocols in the related literature (Ullán et al., 2013; Sauer et al., 2016). All art programs were free for participants. The materials were supplied by the project’s lead researchers and all time was volunteered: one arts instructor volunteer, one art class assistant volunteer, and one art class observer volunteer—reflecting the structure of identical studies (Byrne and MacKinlay, 2012). When each experimental art program began, a maximum size of eight participants per program was set. However, group sizes did fluctuate throughout the research project due to missed sessions (e.g., due to illness) and attrition. Nonetheless, the volunteer to participant ratio was never lower than 1:4. Group size was considered heavily, based on discussions with dementia programming experts and the related literature (Caulfield, 2011; Ullán et al., 2013; Camic et al., 2015; Flatt et al., 2015; Hazzan et al., 2016; Windle et al., 2018b). Further, the volunteers’ focus was to encourage and engage the participants, not their fellow volunteers, to avoid complications noted in similar studies (Byrne and MacKinlay, 2012).

The curriculum was carefully created to support the learning of new skills and understanding, while still stimulating and engaging in order to trigger potential cognitive mechanisms, with input provided by a collaborative group of artists, psychologists, and dementia experts: artist and instructor S. Wiseheart; artist, professor, and experimental psychologist M. Wiseheart; arts researcher and experimental psychologist A. D’Souza; dementia expert A. Ubell; and artist, instructor, and arts and dementia researcher K. Johnson. Each element is introduced in an intentional order; from the use of space, to using color and texture to fill that space, to introducing lines into that space, to using lines to create shapes, to using shapes to create forms, to using value to fill those forms. Similarly, the principles are introduced in an intentional order: from emphasizing a single element, to a variety of elements, to using a variety of similar elements to create harmony, to using harmonious elements to create movement, to turning that movement into rhythm, to considering the proportions, balance, and gradation of the overall image. Further, each element and principle was paired together to highlight and illustrate each other in a coherent and understandable way: using space to create emphasis; using a variety of colors; creating harmony with similar textures; creating movement with lines; creating rhythm with repeated shapes; creating proportion with different sized forms; creating balance with forms; and creating gradual change with values.

In tune with art’s advantageous flexibility, programs were created in order to foster creativity and enable participation malleability. A key aspect of the project’s intervention was its suitability for those with varying types and severities of dementia as well as those with varying artistic backgrounds. To ensure flexibility and realistic applications, participants who arrived late, needed breaks, or wished to leave early or stay late were not excluded from the study. While participants were guided and instructed regarding each week’s activity, novel creativity was never halted, instead it was encouraged and adapted into the program. Participants were able to decide on their level of engagement in the program, ranging from sitting with the group while being exposed to the art lessons, art supplies, learning tools, group discussions and imagery, to doing all that while creating multiple artworks each day and showing their work at the end of the class. This is in line with the related literature, with a focus on supporting participant growth, success, individuality, and personhood; highlighting participants’ active contributions and strengths as opposed to emphasizing their limitations (Ullán et al., 2013; Flatt et al., 2015; Sauer et al., 2016; Schneider et al., 2019).

Materials were carefully selected: markers, pencil crayons, stickers, and glue sticks were non-toxic; material sizes were considered and small objects were avoided to ensure a safe program; poster board was used instead of standard paper for collage activities to provide more stability for the artworks; and textured mazes were used to illustrate the terms “movement” and “line,” with tape creating the lines on the page for a textural cue.

While the experimental participants were engaged with the art training program, the active control participants were offered their respective location’s usual activities (e.g., group singing, exercises, independent activities such as reading or knitting, etc.) following suggested guidelines (Young, 2014) and practice (Rusted et al., 2006).

### Testing

Before and after the art programs or control activities, participants completed assessments. The WAIS-IV Backward Digit Span (Rankin et al., 2007; Fernandez-Duque and Black, 2008; Huntley and Howard, 2010) was used to measure working memory and the Montreal Cognitive Assessment (MoCA) (Nasreddine et al., 2005) was used to measure overall cognitive function^6^ ^,7^. The MoCA’s delayed recall subsection was also used to measure delayed recall. The tasks used are validated and reliable quantitative measures selected based on dementia expert feedback and the assessments’ appropriateness for people with varying dementia types (Folstein et al., 1975; Nasreddine et al., 2005; Rankin et al., 2007; Fernandez-Duque and Black, 2008; Huntley and Howard, 2010; Matthews, 2016; Windle et al., 2018a). Although infrequent, if a participant was unavailable or uninterested in completing a task session 1 day, their session was rebooked when possible and attempted again.

### Volunteers

Twenty-six volunteers assisted with the research project. All volunteers were blind to participant condition and study hypotheses^8^. Volunteers were interviewed, reference-checked, vulnerable sector checked, tuberculosis tested, and carefully trained with multiple training sessions, with instructors and testers attending mandatory knowledge assessment sessions before beginning to volunteer. All full-time volunteers were provided with a dementia orientation by an expert in the field before they began volunteering, while substitute volunteers were provided with an introductory visit and tour of an assisted-living building before volunteering.

### Outcome Evaluation

Pre-testing occurred the week of and the week before each respective art program began, observations occurred during each respective program and testing session, and post-testing occurred the week of and the week after each respective art program ended. Task data were recorded by one primary volunteer data recorder and three secondary data recorder volunteers, with an inter-rater reliability between the primary and secondary data recorders averaging above 90% for each quantitative task. Where non-identical, task scores were averaged. All data entry volunteers were provided with training before beginning data entry.

Data analyses were completed using JASP, a graphical user interphase (GUI) for R (Fu et al., 2020). Qualitative analyses investigating the behavioral and psychological effects of art training were also recorded by the primary data recorder and were coded using thematic analysis, but are not reported here (reported at https://osf.io/dpc82/).

The results of the MoCA and Digit Span were analyzed with an independent samples *t*-test, assessing the differences between the experimental and control group by comparing the groups’ difference score means (post—pre). Independent samples *t*-tests were also completed for baseline MoCA, Digit Span, and MMSE scores to ensure that both groups were comparable at baseline^9^. All tasks met this requirement. Bayesian statistics, which are able to provide support for both null and experimental hypotheses, were used. Data are available at https://osf.io/dpc82/.

## Results

### Choice of Prior

The present study used Bayesian analyses. Before beginning the study, we chose a prior that reflected our belief at the time that art training should produce a small effect on cognition, and our predictions matched this belief (i.e., less cognitive decline should occur in the experimental groups rather than the control groups). Thus, as an informed prior, we initially planned to use a normal distribution with a mean of 0.3 and a *SD* of 0.5. However, since finishing the study, several reviews of the training literature have been published and it has become evident that training programs are unlikely to produce far transfer: a second-order meta-analysis of training programs failed to show far transfer effects, across a wide range of training domains (Sala et al., 2019). Therefore, we have chosen to use a normal distribution with a mean of zero and a *SD* of 0.5 as our prior in reported analyses, since that reflects our beliefs after reading recent meta-analyses. Use of our initial prior resulted in slightly but not meaningfully greater evidence for null results.

### Sample Size Justification

Using the SSDbain package for R (Fu et al., 2020), we computed the number of participants that would be needed to have 80% power to detect an effect size of *d* = 0.3 with a BF_10_ threshold of 3. With a hypothetical sample size of 317, we would reach 80% power for BF_10_ and 96% power for BF_01_. However, with the challenges associated with recruiting those with dementia, in addition to the present study’s rigorous methodology, this number of participants could not be recruited. Nonetheless, the present study’s improvement on previous art and dementia studies (Locher, 2007; Chancellor et al., 2014; Windle et al., 2014, 2018a,b; Young et al., 2015; Matthews, 2016; Sauer et al., 2016), as well as its contextually large sample size [*n* = 39 (Hattori et al., 2011) and *n* = 21 (Rusted et al., 2006)], support the importance of its completion and value of its conclusions amongst the comparable data.

### Pilot Project

A pilot was completed prior to beginning the preset study (Matthews, 2016). The pilot assessed the cognition of two groups of people with dementia: an experimental art training group (*n* = 9), who participated in an 8-week visual art training program, and a waitlist control group (*n* = 6), who participated in their usual dementia center activities. The results of the pilot study suggested a possible improvement in working memory as a result of art training (as measured by Backward Digit Span post—pre difference scores; experimental, *M* = 1.3, *SD* = 1.7; waitlist control, *M* = −0.3, *SD* = 0.5). However, the pilot’s sample size was too small to draw firm conclusions, with a Bayesian *t*-test indicating an indeterminate difference between groups, *BF*_10_ = 1.86. The pilot study provided information regarding the feasibility and potential of future larger-scale art training projects and laid the groundwork for the present study’s duration, measures, training curriculum, and recruitment.

### Baseline Characteristics: Background Questionnaire

Participant background characteristics, including sex and age information, are reported in Table 2. Baseline MMSE scores documented participant dementia severity according to their surrogate Clinical Dementia Rating Scale scores: 26–29 questionable dementia, 21–25 mild dementia, 11–20 moderate dementia, 0–10 severe dementia (Perneczky et al., 2006). Any individual falling within the questionable range was cross-referenced with their questionnaire data to confirm diagnosis. The experimental (*M* = 13.6; *SD* = 7.1) and control groups (*M* = 13.3; *SD* = 6.6) were deemed comparable based on having nearly identical MMSE scores, even though statistical evidence for equivocal scores was weak, BF_10_ = 0.487, *d* = 0.044 [−0.49, 0.58]. There were a mix of dementia types included in the study, including Alzheimer’s disease and vascular dementia^10^, and the severity of dementia for both groups ranged from mild to severe.

**TABLE 2:** Participant demographic information.

	**N**	**Sex**	**MMSE (SD)**	**Dementia severity**	**Dementia type**	**Age^b^ (SD)**	**Age range^b^**
Experimental	Overall: 27	F: 16	13.6 (7.1)	Questionable: 1	Unprovided: 6	79.7 (8.9)	54–90
	AL:12	M: 11		Mild: 6	AD: 6		
	DP:15			Moderate: 8	Unspecified: 15		
				Severe: 12			
Control	Overall: 26^a^	F: 22	13.3 (6.6)	Questionable: 1	Unprovided: 10	82.3 (8.4)	66–96
	AL:8	M: 4		Mild: 3	AD: 7		
	DP:18			Moderate: 13	AD and Vascular: 1		
				Severe: 9	Vascular: 1		
					Unspecified: 7		

*^*a*^One participant did not complete the Digit Span. ^*b*^In years; 6 experimental and 8 control participants did not provide age information. AL, Assisted-Living Retirement; DP, Day Program; M, Male; F, Female; AD, Alzheimer’s disease.*

### Overall Cognition: MoCA

There is weak evidence that overall cognition, measured by MoCA post—pre difference scores, did not differ between the experimental (*M* = 0.37; *SD* = 2.7) and waitlist control groups (*M* = 0.23; *SD* = 3.2), BF_10_ = 0.487, *d* = 0.047 [−0.49, 0.59]. There is weak evidence that pre-test scores also did not differ between groups, BF_10_ = 0.496, *d* = 0.076 [−0.46, 0.62]. Thus, it is twice as likely that both groups performed identically on the MoCA than that they performed differently, which suggests that overall cognition was not affected by 8 weeks of visual art training. Pre- and post-test results for the MoCA can be found in Table 3.

**TABLE 3:** Pre- and Post-test scores for each task according to original assigned group.

	**Experimental group**		**Waitlist control group**	
	**Pre-test mean (SD)**	**Post-test mean (SD)**	**Pre-test mean (SD)**	**Post-test mean (SD)**
MMSE	13.6 (7.1)	NA (NA)	13.3 (6.6)	NA (NA)
MoCA	8.8 (7.5)	9.2 (8.0)	8.3 (5.7)	8.5 (5.9)
MoCA delayed recall	0.50 (1.1)	0.41 (1.3)	0.0 (0.0)	0.15 (0.61)
Backward Digit Span	2.6 (2.4)	2.6 (2.4)	2.7 (2.6)	2.8 (2.8)

### Delayed Memory: MoCA Delayed Recall

Delayed recall was measured by MoCA delayed recall post—pre difference scores. Based on our findings, it is indeterminate whether delayed recall differed between experimental (*M* = −0.093; *SD* = 0.68) and waitlist control groups (*M* = 0.15; *SD* = 0.61), BF_10_ = 1.003, *d* = −0.38 [−0.92, 0.17]. Performance was at or near floor on this task, making it impossible to interpret results. Pre- and post-test results for the MoCA’s delayed recall subsection can be found in Table 3.

### Working Memory: Digit Span

There is weak evidence that working memory, measured by Backward Digit Span post—pre difference scores, did not differ between the experimental (*M* = −0.037; *SD* = 1.3) and waitlist control group (*M* = 0.040; *SD* = 1.8), BF_10_ = 0.491, *d* = −0.049 [−0.59, 0.50]. There is weak evidence that pre-test scores did not differ between groups, BF_10_ = 0.492, *d* = −0.051 [−0.60, 0.49]. Thus, it is twice as likely that both groups performed identically on the Backward Digit Span than that they performed differently, which suggests that working memory was not affected by 8 weeks of visual art training. Pre- and post-test results for the Backward Digit Span can be found in Table 3.

## Discussion

We found no quantitative benefits from 8 weeks of arts training on overall cognition, working memory, or delayed recall. These results suggest that previous RCT failures to find cognitive or memory benefits from arts interventions were not due to their specific test choices, as we have replicated these studies using alternate measures. Further, we used a strong arts training intervention designed by a team of experts in art, dementia, and teaching, making it unlikely that lack of intervention quality explains our null findings.

While the present study did not find quantifiable cognitive improvements for those who participated in arts training, it is important to highlight the achievement of the study’s mere completion and the artistic potential of dementia patients. The present study provided an arts training program to multiple dementia care locations, successfully providing an educational experience to those with varying types and severities of dementia. The program provided an opportunity for student volunteers to support, teach, and learn from the participants they encountered, providing a gateway for people in the community to help and engage with individuals in the dementia care setting—and vice versa. The program provided the means for participants to learn, create, and share, all while keeping their creations at no cost to them. Regardless of cognitive effects, the art program provided an opportunity for even the most severely compromised participant to engage, create, and share with their peers and members of their community.

While our quantifiable measures found no cognitive effects, it is understandable how studies utilizing qualitative analyses have supported art’s cognitive impact: by participating in art, learning about terms and techniques, completing art projects and/or engaging with your peers in art discussions, are these participants not engaging in a cognitive exercise?^11^ While these effects may not be seen at post-testing with quantitative measures, or perhaps at all via quantitative analysis, future research should explore the immediate quantifiable effects of arts interventions.

The present study addressed the limitations of the pilot project, where a potential working memory benefit was found but was unsubstantiated based on the pilot’s small sample size. Utilizing the knowledge gained from the pilot study, including refining the task battery and more than tripling the sample size, the present study found a null effect for working memory differences between groups. Thus, we conclude that the pilot’s possible working memory benefit was a false positive resulting from the pilot’s small sample size, emphasizing the importance of having sufficient sample size within dementia RCTs.

A limitation of the present study is the lack of dementia type information. Although dementia type was requested, multiple reports were left either unanswered or answered with a general “dementia” diagnosis. This limitation is one seen throughout the dementia literature, including the art-dementia literature (Kahn-Denis, 1997; Rentz, 2002; Stewart, 2004; Kinney and Rentz, 2005; Camic et al., 2014, 2015; Young, 2014; Flatt et al., 2015; Young et al., 2015; Hazzan et al., 2016), and represents the typical situation within a dementia care environment which includes individuals of varying dementia types. This limitation may be mediated in the future by recruiting from hospital participant pools, where diagnosis would be recorded upon entry, or with better research infiltration into other dementia care locations. For example, if dementia care locations with an interest in research had potential participants screened upon entry, it would enable access to interested persons’ diagnosis information at the onset of a research project. This would also potentially provide a greater participant pool, addressing the need for dementia studies with greater sample sizes.

Readers might believe that we failed to examine measures that would show intervention effects. It is entirely possible that a different set of measures would show benefits. However, we believe it extremely unlikely that quantifiable cognitive benefits will occur from art training in a dementia population; two previous studies plus this study failed to show memory benefits utilizing quantitative measures (Rusted et al., 2006; Hattori et al., 2011), and a second-order meta-analysis demonstrated a lack of far transfer training benefits regardless of domain or task (Sala et al., 2019). Based on these findings, we are confident that our selected measures are not the cause of our null results.

## Conclusion

When a person develops the symptoms of dementia, loss is inevitable, but that does not mean that loss is all there is. People with dementia often wish to participate in activities, to be a part of something and feel valued, and this wish is not an impossibility. While cognition may be unaffected by art training for those with dementia, the present study remains supportive of dementia arts interventions based on the intrinsic value of learning and engaging in art. Art training programs can bring together the community, both by creating a community within each individual classroom as well as joining those from inside and outside the dementia care location. Art programs provide an easily implemented and patient-accessible program for dementia care locations, utilizing simple low-cost materials, adaptable procedures, and no-cost volunteers. The present study demonstrates the feasibility of arts programming for dementia patients, as well as a testament to the abilities of those with the disease.

## Data Availability Statement

The dataset generated for this study can be found at https://osf.io/dpc82/.

## Ethics Statement

The studies involving human participants were reviewed and approved by the York University Human Participants Research Sub-Committee. Substitute written informed consent was provided by a potential participant’s care location via written consent for the project along with a list of approved individuals who could participate. Participants provided verbal assent.

## Author Contributions

KJ designed the intervention, which was refined by AD’S and MW. KJ recruited participating locations, randomized participants, assigned participants to their respective group, coordinated the intervention and testing sessions, and conducted qualitative analyses. MW ran quantitative analyses. KJ wrote the manuscript with assistance from AD’S and MW. All authors selected and designed the testing battery.

## Conflict of Interest

The authors declare that the research was conducted in the absence of any commercial or financial relationships that could be construed as a potential conflict of interest.

## Footnotes

1 Alzheimer’s disease (Kahn-Denis, 1997; Fornazzari, 2005), vascular or multi-infarct dementia (depending upon where the stroke-related lesions occurred; Stewart, 2004), and fronto-temporal dementia (Kleiner-Fisman and Lang, 2004; Fornazzari, 2005).

2 The majority of the literature focuses on either an unspecified type of dementia or a specified mixture of dementia types (Kahn-Denis, 1997; Rentz, 2002; Stewart, 2004; Kinney and Rentz, 2005; Rusted et al., 2006; Byrne and MacKinlay, 2012; Ullán et al., 2013; Camic et al., 2014, 2015; Young, 2014; Flatt et al., 2015; Young et al., 2015; Sauer et al., 2016; Windle et al., 2018b; Schneider et al., 2019).

3 The minimum required attendance was 50% or more classes or participating in at least 4 of 8 weekly lessons, which was our attempt to ensure sufficient dose of intervention while maintaining most participants in the sample. Removing the nine participants whose attendance was less than 75% of classes did not meaningfully change effect sizes, which remained near zero.

4 The visual stimuli of the MMSE were enlarged and shown one at a time based on population and location requirements. In addition, the orientation section reflected the locations of the study (Canadian in non-hospital locales).

5 Inclusion criteria for each recruited location included the ability for participants to understand and communicate in English—to varying fluencies—so that they could take part in the assent process, testing questions, and lessons provided during art programming.

6 The visual stimuli of the MoCA were enlarged and shown one at a time based on population and location requirements.

7 The body part pointing task (Matthews, 2016) was also utilized. However, due to the resulting high number of binary results (with participants either scoring 100 or 0%) the task’s results were largely meaningless and will not be reported here. For details, see a fuller description of the results (Johnson, 2018).

8 Both groups received the same assessments and volunteers were not aware of when interventions occurred. Study hypotheses were not revealed to volunteers until their time on the project was concluded.

9 MMSE scores were used for dementia diagnosis confirmation purposes as well as group matching comparison.

10 While dementia type information was requested, most dementia types were either unspecified or unprovided.

11 For details on this from the present study, see https://osf.io/dpc82/.

## References

[B1] Baddeley A. D. Logie R. H. (1999). “Working memory: the multiple-component model,” in *Models of Working Memory: Mechanisms of Active Maintenance and Executive Control*, eds Miyake A. Shah P. (Cambridge: Cambridge University Press), 28–61. 10.1017/CB09781139174909.005

[B2] Bhattacharya J. Petsche H. (2002). Shadows of artistry: cortical synchrony during perception and imagery of visual art. *Cogn. Brain Res.* 13 179–186. 10.1016/S0926-6410(01)00110-0 11958960

[B3] Byrne L. MacKinlay E. (2012). Seeking meaning: making art and the experience of spirituality in dementia care. *J. Religion Spirituality Aging* 24 105–119. 10.1080/15528030.2012.633416

[B4] Camartin K. (2015). Art therapy in a psychogeriatric unit. *Advocate* 22 20–21.

[B5] Camic P. M. Baker E. L. Tischler V. (2015). Theorizing how art gallery interventions impact people with dementia and their caregivers. *Gerontologist* 56 1033–1041. 10.1093/geront/gnv063 26185152

[B6] Camic P. M. Tischler V. Pearman C. H. (2014). Viewing and making art together: a multi-session art-gallery-based intervention for people with dementia and their carers. *Aging Mental Health* 18 161–168. 10.1080/13607863.2013.818101 23869748

[B7] Caulfield S. (2011). “Art, museums, and culture,” in *Enhancing Cognitive Fitness in Adults*, eds Hartman-Stein P. E. La Rue A. (New York, NY: Springer), 301–323.

[B8] Chancellor B. Duncan A. Chatterjee A. (2014). Art therapy for Alzheimer’s disease and other dementias. *J. Alzheimer’s Dis.* 39 1–1. 10.3233/JAD-131295 24121964

[B9] Cowl A. L. Gaugler J. E. (2014). Efficacy of creative arts therapy in treatment of Alzheimer’s disease and dementia: a systematic literature review. *Activities Adaptation Aging* 38 281–330. 10.1080/01924788.2014.966547

[B10] Douglas S. James I. Ballard C. (2004). Non-pharmacological interventions in dementia. *Adv. Psychiatric Treat.* 10 171–177. 10.1192/apt.10.3.171

[B11] Stewart E (2004). Art therapy and neuroscience blend: working with patients who have dementia. *Art Ther.* 21 148–155. 10.1080/07421656.2004.10129499

[B12] Ehresman C. (2014). From rendering to remembering: art therapy for people with Alzheimer’s disease. *Int. J. Art Ther.* 19 43–51. 10.1080/17454832.2013.819023

[B13] Fernandez-Duque D. Black S. E. (2008). Selective attention in early dementia of Alzheimer type. *Brain Cogn.* 66 221–231. 10.1016/j.bandc.2007.08.003 17950510

[B14] Flatt J. D. Liptak A. Oakley M. A. Gogan J. Varner T. Lingler J. H. (2015). Subjective experiences of an art museum engagement activity for persons with early-stage Alzheimer’s disease and their family caregivers. *Am. J. Alzheimer’s Dis. Other Dement.* 30 380–389. 10.1177/1533317514549953 25216658 PMC4362745

[B15] Folstein M. F. Folstein S. E. McHugh P. R. (1975). “Mini-mental state”: a practical method for grading the cognitive state of patients for the clinician. *J. Psychiatric Res.* 12 189–198. 10.1016/0022-3956(75)90026-6 1202204

[B16] Fornazzari L. R. (2005). Preserved painting creativity in an artist with Alzheimer’s disease. *Eur. J. Neurol.* 12 419–424. 10.1111/j.1468-1331.2005.01128.x 15885044

[B17] Foster C. (2006). *Vocabulary.* Available online at: www.oberlin.edu/amam/asia/sculpture/documents/vocabulary.pdf (accessed November 24, 2014).

[B18] Fu Q. Hoijtink H. Moerbeek M. (2020). Sample-size determination for the Bayesian t test and Welch’s test using the approximate adjusted fractional Bayes factor. *Behav. Res.* 10.3758/s13428-020-01408-1 32632740 PMC7880954

[B19] Hattori H. Hattori C. Hokao C. Mizushima K. Mase T. (2011). Controlled study on the cognitive and psychological effect of coloring and drawing in mild Alzheimer’s disease patients. *Geriatrics Gerontol. Int.* 11 431–437. 10.1111/j.1447-0594.2011.00698.x 21518170

[B20] Hazzan A. A. Humphrey J. Kilgour-Walsh L. Moros K. L. Murray C. Stanners S. (2016). Impact of the ‘Artful Moments’ intervention on persons with dementia and their care partners: a pilot study. *Can. Geriatrics J.* 19:1. 10.5770/cgj.19.220 27403209 PMC4922369

[B21] Hersch E. C. Falzgraf S. (2007). Management of the behavioral and psychological symptoms of dementia. *Clin. Intervent. Aging* 2:611. 10.2147/CIA.S1698 18225462 PMC2686333

[B22] Huntley J. D. Howard R. J. (2010). Working memory in early Alzheimer’s disease: a neuropsychological review. *Int. J. Geriatric Psychiatry J. Psychiatry Late Life All. Sci.* 25 121–132. 10.1002/gps.2314 19672843

[B23] Johnson K. (2018). *The Effect of Art Training on Dementia.* Doctoral Dissertation, York University,Toronto, ON.

[B24] Kahn-Denis K. B. (1997). Art therapy with geriatric dementia clients. *Art Ther.* 14 194–199.

[B25] King J. Goeman D. Koch S. (2015). Dementia care in the community: access for culturally and linguistically diverse communities. *Alzheimer’s Dement. J. Alzheimer’s Assoc.* 11:581 10.1016/j.jalz.2015.06.772

[B26] Kinney J. M. Rentz C. A. (2005). Observed well-being among individuals with dementia: memories in the Making©, an art program, versus other structured activity. *Am. J. Alzheimer’s Dis. Other Dement.* 20 220–227. 10.1177/153331750502000406 16136845 PMC10833299

[B27] Kleiner-Fisman G. Lang A. E. (2004). Insights into brain function through the examination of art: the influence of neurodegenerative diseases. *Neuroreport* 15 933–937. 10.1097/00001756-200404290-00001 15076710

[B28] Locher P. J. (2007). Editorial: twenty-five years of empirical studies of the arts. *Empirical Stud. Arts* 25 117–120. 10.2190/G055-5326-0264-0786 22612255

[B29] Matthews K. (2016). *The Effect of Art Training on Dementia.* Masters thesis, York University, Toronto, ON.

[B30] Mendez M. F. (2004). Dementia as a window to the neurology of art. *Medical Hypotheses* 63 1–7. 10.1016/j.mehy.2004.03.002 15193339

[B31] Miller B. L. Hou C. E. (2004). Portraits of artists: emergence of visual creativity in dementia. *Arch. Neurol.* 61 842–844. 10.1001/archneur.61.6.842 15210520

[B32] Nasreddine Z. S. Phillips N. A. Bédirian V. Charbonneau S. Whitehead V. Collin I. (2005). The montreal cognitive assessment, MoCA: a brief screening tool for mild cognitive impairment. *J. Am. Geriatrics Soc.* 53 695–699. 10.1111/j.1532-5415.2005.53221.x 15817019

[B33] Parsa A. Humble L. Gerber C. (2010). Two art museum programs for people with dementia. *Museums Soc. Issues* 5 217–234. 10.1179/msi.2010.5.2.217

[B34] Peisah C. Lawrence G. Reutens S. (2011). Creative solutions for severe dementia with BPSD: a case of art therapy used in an inpatient and residential care setting. *Int. Psychogeriatr.* 23 1011–1013. 10.1017/S1041610211000457 21426619

[B35] Pérez-Fabello M. J. Campos A. (2007). Influence of training in artistic skills on mental imaging capacity. *Creativity Res. J.* 19 227–232. 10.1080/10400410701397495

[B36] Perneczky R. Wagenpfeil S. Komossa K. Grimmer T. Diehl J. Kurz A. (2006). Mapping scores onto stages: mini-mental state examination and clinical dementia rating. *Am. J. Geriatric Psychiatry* 14 139–144. 10.1097/01.JGP.0000192478.82189.a8 16473978

[B37] Perry R. J. Hodges J. R. (1999). Attention and executive deficits in Alzheimer’s disease: a critical review. *Brain* 122 383–404. 10.1093/brain/122.3.383 10094249

[B38] Rankin K. P. Liu A. A. Howard S. Slama H. Hou C. E. Shuster K. (2007). A case-controlled study of altered visual art production in Alzheimer’s and FTLD. *Cogn. Behav. Neurol.* 20:48. 10.1097/WNN.0b013e31803141dd 17356345 PMC2651227

[B39] Rentz C. A. (2002). Memories in the Making©: Outcome-based evaluation of an art program for individuals with dementing illnesses. *Am. J. Alzheimer’s Dis. Other Dement.* 17 175–181. 10.1177/153331750201700310 12083348 PMC10833850

[B40] Rusted J. Sheppard L. Waller D. (2006). A multi-centre randomized control group trial on the use of art therapy for older people with dementia. *Group Analysis* 39 517–536. 10.1177/0533316406071447

[B41] Sala G. Aksayli N. D. Tatlidil K. S. Tatsumi T. Gondo Y. Gobet F. (2019). Near and far transfer in cognitive training: a second-order meta-analysis. *Collabra Psychol.* 5 1–22. 10.1525/collabra.203 33021500

[B42] Sauer P. E. Fopma-Loy J. Kinney J. M. Lokon E. (2016). “It makes me feel like myself”: person-centered versus traditional visual arts activities for people with dementia. *Dementia* 15 895–912. 10.1177/1471301214543958 25049353

[B43] Schneider J. Hazel S. Morgner C. Dening T. (2019). Facilitation of positive social interaction through visual art in dementia: a case study using video-analysis. *Ageing Soc.* 39 1731–1751. 10.1017/S0144686X1800020X 31285637 PMC6614029

[B44] ShinagawaS.MillerB. L. (2014). Dementia and art. * 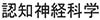 * 15 171–174. 10.11253/ninchishinkeikagaku.15.171

[B45] Takahashi J. Hatakeyama T. (2011). Spatial and nonspatial working memory and visual search. *Psychol. Rep.* 108 893–907. 10.2466/04.22.24.PR0.108.3.893-907 21879636

[B46] Tuokko H. Vernon-Wilkinson R. Weir J. Beattie B. L. (1991). Cued recall and early identification of dementia. *J. Clin. Exp. Neuropsychol.* 13 871–879. 10.1080/01688639108405104 1779027

[B47] Ullán A. M. Belver M. H. Badía M. Moreno C. Garrido E. Gómez-Isla J. (2013). Contributions of an artistic educational program for older people with early dementia: An exploratory qualitative study. *Dementia* 12 425–446. 10.1177/1471301211430650 24336953

[B48] Van Buren B. Bromberger B. Potts D. Miller B. Chatterjee A. (2013). Changes in painting styles of two artists with Alzheimer’s disease. *Psychol. Aesthetics Creativity Arts* 7:89 10.1037/a0029332

[B49] Windle G. Gregory S. Howson-Griffiths T. Newman A. O’Brien D. Goulding A. (2018a). Exploring the theoretical foundations of visual art programmes for people living with dementia. *Dementia* 17 702–727. 10.1177/1471301217726613 28914090 PMC6068961

[B50] Windle G. Gregory S. Newman A. Goulding A. O’Brien D. Parkinson C. (2014). Understanding the impact of visual arts interventions for people living with dementia: a realist review protocol. *Syst. Rev.* 3:91. 10.1186/2046-4053-3-91 25128286 PMC4141269

[B51] Windle G. Joling K. J. Howson-Griffiths T. Woods B. Jones C. H. van de Ven P. M. (2018b). The impact of a visual arts program on quality of life, communication, and well-being of people living with dementia: a mixed-methods longitudinal investigation. *Int. Psychogeriatrics* 30 409–423. 10.1017/S1041610217002162 29113610

[B52] Young R. (2014). *The Cognitive Impact of Art-gallery Interventions for People with Dementia.* Doctoral dissertation, Canterbury Christ Church University, Canterbury.

[B53] Young R. Tischler V. Hulbert S. Camic P. M. (2015). The impact of viewing and making art on verbal fluency and memory in people with dementia in an art gallery setting. *Psychol. Aesthetics Creativity Arts* 9:368 10.1037/aca0000030

